# An Investigation of the Socio-Economic Status of the Addicts in Lashar and Nikshahr County and Its Comparison With Ordinary People

**DOI:** 10.5539/gjhs.v7n3p194

**Published:** 2014-11-30

**Authors:** Ahmadali Raeisei, Azizollah Arbabisarjou, Azizollah Mojahed

**Affiliations:** 1 Department of Cardiology, Zahedan University of Medical Sciences, Zahedan, Iran; 2 Pregnancy Health Research Center, Zahedan University of Medical Sciences, Zahedan, Iran; 3 Department of Psychology, Zahedan University of Medical Sciences, Zahedan, Iran

**Keywords:** addiction, the socio-economic status, addicted individuals, healthy individuals

## Abstract

The world today is threatened by great disasters and catastrophes and one of the greatest of them is addiction. Addiction is a disaster that threatens all age and sex groups. For instance, in our country more than 2% of people are addicted. In this study, two groups of addicted (170) and healthy (167) individuals that had been selected in the systematic random method, were investigated in terms of the socio-economic status. The data was collected through the questionnaire. The average age and education level were 34.8 and 4.22 respectively among the addicted and 31.27 and 6.3 respectively among the healthy individuals. 83.1% of the addicted and 74.7% of the healthy individuals were married. A significant difference was observed between the education level and addiction with the p=0 using the t-test. A significant relationship was observed between the existence of addiction in the family with the p=0 and addiction among friends and addiction with the p=0.0001 and between job and addiction with the p=0.0115 and between addiction and the level of income with the p=0.0065.

## 1. Introduction

Drug abuse is a common phenomenon all over the world and as the most important social disorder, it has invaded the human community (Bakhshi Poor, 2004; Seyam, 2006). Drug abuse refers to a non-adaptive pattern of drug use that leads to recurrent problems and disadvantages and contains a set of cognitive, behavioral and psychological symptoms.

For every human community, addiction causes damages that contain economic, social, cultural, political and human aspects. Addiction of the younger people in fact leads to the complete destruction and degradation of that community. In many countries, the age of vulnerability to addiction has been stated to range between 20 and 34 years (Madadi & Nogani, 2004; Chen & Wang, 2006).

Every country strongly emphasizes the health of the youth. Unfortunately, there has been a considerable increase in the use of drugs, alcohol and tobacco, specifically among the youth. The studies show that the medical staff experiences a higher level of dependence on the drugs, compared with the other occupations (Shyangwa et al., 2007; Hamermesh & Slemrod, 2008).

The numbers and statistics show great losses of life and property resulting from addiction. Economic costs, death, suicide, heavy crimes and unsuccessful marriages are somehow related to the drug use (Yassini & Rafati, 2009; Grange et al., 2005). Furthermore, adverse social complications, behavioral and psychiatric problems and the risk of medical conditions such as AIDS and Hepatitis, increase as a result of misuse of these drugs. As the factors causing addiction are numerous, complete identification of these factors in every region will help to the effectiveness of prevention activities. On the other hand, early identification of populations at risk and drug abusers is, so as to develop prevention and treatment programs is highly important (Bakhshi Poor, 2004; Davis et al., 2002).

Extensive studies have been conducted regarding the factors involved in the addiction of individuals; for instance, Feyzollahi has explored and examined the social factors related to addiction among the youth in Ilam Province. In this study, the findings indicate that family damages such as family turmoil, existence of family background, parents’ incongruent management and low attachment to the family on the one hand and the destructive effects of the level of uncontrolled opportunities of the individual and negative features of the group of criminal friends and poor educational environment on the other hand, positively affect the individual’s tendency to education (Feyzollahi, 2008); in his study, Nasiri has explored and evaluated the relationship between geographical distribution of housing of low income groups and frequency of addiction in different regions of the city of Babol. In this study, he shows that in southern and marginal areas in the city, improper residential buildings and addiction are more frequent. In this study, the level of dependence of addiction on housing shortage has been determined to be 69% (Nasiri, 2006).

In his thesis entitled “investigation of the effects of socio-economic factors on addiction of the addicts in Kerman rehabilitation center”, Saeid shows that there is a significant relationship between job, parents’ criminal background, the father’s education and socio-economic status of the family, and addiction (Saeid, 2006).

Laoniramai et al. conducted a study regarding the drug use among the youth in Bangkok. This study intended to investigate the effects of population factors, financial status, social base, awareness regarding the drugs, misuse of the drugs and life skills in order to prevent from drug abuse by the youth. The sample population included 354 of the youth aged between 12 and 22 who lived on the margins of Bangkok. The study showed that among the sampled people, 7% had previously used drugs. 4% had never used drugs but 20% had friends that had used drugs at least a couple of times. 11% of them had friends that still used drugs. The main drug used by them was crystal meth (known in Iran as “shisheh”. In total, the people included in the study had little awareness regarding the drug abuse, specifically its symptoms, side effects and punishments resulting from that. Most people knew that drug use is dangerous. The factors that affected the drug use, included: 1) personal factors like monthly income and pension and life skills; 2) family background such as the presence of addicts in the family; and 3) social environment such as the presence of addicts among friends. While studying the life skills of individuals, that were studied as independent factors effective in experiences with the drugs, the researchers realized that spending time with the other members of the family and family members who have had the experience of drug use is the important factor in preventing from the drug use. Allowing children to gain first-hand experiences will help them to increase their life skills. Enhancement of the relationship between the youth and the other members of the family can enhance life skills of children and prevent their addiction (Laoniramai et al., 2005). Winton in 2007 conducted a study on the youth raised in criminal regions in Guatemala City in Latin America. Winton believes that working with the youth that are raised in the so-called helpless families, needs psychological flexibility and different methods must be used for investigation regarding the delinquent youth gangs. He maintains that using participatory methods in such studies is of special importance (Winton, 2007). Most resources available in the country are merely reports of the studies and theories developed by foreign researchers and so far no extensive efforts have been made for careful investigation in socio-economic areas concerning their involvement in addiction of individuals specifically in Lashar district and Nikshahr county. In this regard, the present study intends to investigate the socio-economic status of the addicts in Lashar district and Nikshahr county and its comparison with ordinary people.

## 2. The Research Hypotheses

The effects of economic status are different for addicted and ordinary people.

The effects of social status are different for addicted and ordinary people.

## 3. Methodology

The research method in this study is case-control. The population included all men from villages in Lashar district and Nikshahr County who either had a health care center or were covered by it. The case group or the addicts included 167 individuals and the control group or the healthy people included 170 individuals. In order to conduct the study, the necessary coordination was made with the Presidency of the Health Care System of Nikshahr and subsequently with the help of the workers of care center of Lashar district, a complete list of the addicts in the regions covered by every care center, was provided. The sample size was obtained in the systematic randomized method. For data collecting data, the questionnaire was used. The questionnaire had content validity. Cronbach’s alpha coefficient was used to determine reliability of the questionnaire and it was obtained 0.82. After the data collection, they were coded and entered into the computer. The data analyzed by SPSS version 16 through analysis in descriptive statistics and Chi-square test and t-test.

## 4. Research Findings

167 addicted and 170 healthy individuals were examined. The average age of the addicted individuals was 34.8 years. The lowest age was 15 and the highest age was 78. The average age of healthy individuals was 31.27 and the lowest was 13 and the highest was 74 years. In Table 1 frequency distribution of the study population has been expressed based on the education level and factors for not continuing education.

**Table 1:** Relative frequency distribution of the addicted and healthy individuals based on the education level and the reason for not continuing education

Statistical indexes	Education level					

	Illiterate	Elementary	Guidance	High School	Post-diploma	Higher
Healthy	7.24%	4.19%	8.8%	1.27%	9.2%	1.17%
Addicted	5.33%	2.19%	2.10%	8.7%	4.2%	9.26%
The reason for not continuing education	Lack of interest in the subjects	Expulsion from school	Disease	Lack of tendency of parents	Need	Other factors
Healthy	4.25%	2.17%	3.3%	4.7%	1.13%	6.33%
Addicted	8.37%	6.21%	8.1%	9.9%	2.16%	6.12%

According to the results of table 1, most addicted individuals were illiterate in term of education level (33.5%) and one of the factors for not continuing education, for addicted individuals, is lack of interest in the subjects (37.8%).

According to the results of Table 2, most addicted individuals earned their livings by farming with 43.7% in order to meet the family expenses and their leisure time was spent by being idle (34.7%).

**Table 2:** relative frequency distribution of the ways for meeting the family expenses and how to spend the leisure time (for the addicted and healthy individuals)

Ways for meeting the family expenses	Statistical indexes							

	Farming	Trade	Office work	Parents’ help	Relatives’ help	Free trade	Spouse’s job	Other
Healthy	5.43	1.7%	2.11%	6.17%	5.3%	.	2.1%	9.15%
Addicted	7.43%	2.4%	4.8%	9%	1.7%	4.2%	4.5%	8.19%
How to spend the leisure time	Studying	Sport	Recreation	Unemployment	Friendly gathering		TV	The other
Healthy	5.16%	6.10%	8.18%	6.10%	6.10%		4.12%	6.20%
Addicted	4.5%	4.2%	8.10%	7.34%	7.31%		6.4%	4.10%

### 4.1 Testing the Hypotheses

A: in order to determine the relationship between married and single life and addiction, Chi-square test was conducted and it was observed that there is no significant relationship between these two in statistical terms.

B: in the two concerned groups, the education level was investigated and based on the t-test, it was determined that there is a significant difference between the average of education level in the considered groups with p=0.0; therefore, the average education level among the healthy and addicted individuals was 6.30 and 4.22 respectively.

C: in order to determine the relationship between the background of addiction in the family and addiction, Chi-square test was conducted and a statistical significant relationship between these two with the p=0.00 was confirmed. Therefore, 28% of the healthy individuals and 79% of the addicted individuals had an addicted person or persons in their family.

D: there is a significant statistical relationship between addiction among friends and addiction with p=0.00. Therefore, 53% of the healthy individuals and 88% of the addicted individuals have had addicted friends.

E: there is a significant relationship between earning income (occupation) and addicted groups and ordinary persons with p=0.0115; such that healthy persons earn income by: farming (22%), parents (8.9%), office work (5.6%), trade (3.6%), the relatives’ help (1.8%), the spouse’s work (0.6%) and other (0.8%); yet the addicted individuals do so by: farming (21.7%), parents (4.5%), office work (4.2%), trade (2.1%), relatives’ help (3.6%), the souse’s work (2.7%) and other (9.8%) and free trade (1.2%).

F: in order to investigate the relationship between unemployment and addiction, Chi-square test was carried out and no significant relationship was observed.

G: in order to investigate and determine the relationship between the income level and addiction, Chi-square test was conducted which was observed to have a significant relationship with p=0.0065. Therefore, 34.7% of the healthy individuals had an income lower than 500.000 tomans and 13.6% of them between 510.000 and 1.000.000 tomans and 2.1% of them more than 1.000.0000 but 40.7% of the addicted individuals had lower than 500.000 tomans and 6.5% of them between 510.000 and 1.000.000 and 2.4% of them more than 1.000.000 tomans.

H: there is a significant relationship between how leisure time is spent and addiction with p=0.0; in such a way that healthy individuals mostly spent their leisure time by healthy recreations (9.5%), studying (8.3%), radio and TV (6.2%), sport (5.3%), friendly gatherings (5.3%), being idle (5.3%) and other (10.4%); yet addicted individuals spent their leisure time mostly by being idle (17.2%), friendly gatherings (15.7%), healthy recreations (5.3%), studying (2.7%), radio and TV (2.1%), sport (1.2%) and other (5.3%).

## 5. Discussion and Conclusion

The average age of the addicted individuals was more than that of non-addicted individuals. The maximum and minimum age of addicted individuals was more than non-addicted ones. Most addicted individuals were in the age group of 29-47 years and most non-addicted individuals were in the age group lower than 29 years and this might show the fact that addicted individuals have suffered more in their lives and that most have faced more difficulties which have led them to addiction. Average education level of addicted individuals was lower than that of the non-addicted individuals.

The t-test also states a significant statistical difference between the two groups in such a way that the highest difference is related to the high school time. As the studies show, the students reach the level of perception and awareness, to make their own decisions and, as stated by the psychologists, to have abstract thinking, for the first time in the high school period. The increase of education refers to the increase of the level of awareness and perception of the individual; it might be due to the same reason that non-addicted individuals with higher education levels have had fewer tendencies for addiction. The research results showed that the relationship between non-addicted individuals and their teachers has been in total better than that of addicted individuals and also that healthy individuals have less often stopped their education (66.8% compared with 91.9%); also, addicted individuals have stopped education earlier than non-addicted individuals. Teachers play a highly significant role in the education and training of students in the society and if the relationship between these two groups is intimate and close and there is a mutual sense of responsibility and commitment by teachers and relatives and gratitude by students, students can be expected to take steps in the right path and become responsible in the future (Raeisi, 1999; Arevalo et al., 2008). The results obtained are in line with the findings by Karimi (1999) who has considered the role of education to be effective in preventing from addiction.

86.2% of the addicted individuals and 92.9% of the non-addicted individuals have lived through their childhood with their parents. No one can deny the important educational and constructive role of the family. The numbers above show that addicted individuals, more than healthy individuals, have been deprived of the affection of parents, friendly and intimate family atmosphere and its educational role; and this might have been effective in their tendency to addiction. The average number of the family members of these two groups is close but regarding the addicted individuals, it is slightly more. 83.1% of addicted individuals and 74.7% of the healthy individuals were married; that is to say, addicted individuals have been married more than healthy individuals. 79.5% of the addicted individuals and 67.3% of the healthy individuals had children. 20.4% of the addicted individuals and 10.7% of the healthy individuals felt dissatisfied with their family atmosphere. The results obtained show that the turmoil and tension within the family might be more among addicted individuals than healthy ones; therefore, a family aura of tension and turmoil with the spouses and children, might lead the individual to addiction in order to escape this situation.

64.2% of the addicted individuals and 57.1% of the healthy individuals are unemployed; that is to say, there were more unemployed addicted individuals than unemployed healthy individuals; however, by carrying out the Chi-square test, no significant relationship was obtained between them.

In general, married life, low education level, not going the through educational period with proper conditions and lack of good and proper relationship with the teachers, not continuing education, not living with the parents in childhood and dissatisfaction with the family atmosphere can all be obtained from the risk-factors of addiction. Furthermore, the parents’ death, low income and high costs, not having a proper job, physical and psychological diseases, not having healthy recreations leisure activities, not having healthy sport facilities, lack of faith and its weakness, making wrong friends and also presence of addicted individuals in the family and the relatives can be considered as the factors of addiction in Lashar district and Nikshahr county.
